# Single-Dose Rifampicin Leprosy Chemoprophylaxis for Household Contacts in Kiribati: An Audit of a Combined Retrospective and Prospective Approach

**DOI:** 10.3390/tropicalmed9030058

**Published:** 2024-03-01

**Authors:** Patrick O. Campbell, Temea Bauro, Erei Rimon, Eretii Timeon, Caitlin Bland, Nabura Ioteba, Nicholas M. Douglas, Arturo Cunanan, Stephen T. Chambers

**Affiliations:** 1 Department of Pathology and Biomedical Science, University of Otago, Christchurch 8011, New Zealand; steve.chambers@otago.ac.nz; 2 Department of Infectious Diseases, Christchurch Hospital, Te Whatu Ora Waitaha, Canterbury 8011, New Zealand; nick.douglas@otago.ac.nz; 3 Government of the Republic of Kiribati Ministry of Health and Medical Services, Tarawa P.O. Box 268, Kiribati; temea.bauro@mhms.gov.ki (T.B.);; 4 Otago Medical School, University of Otago, Christchurch 8011, New Zealand; 5 Pasifika Medical Association, Christchurch 8011, New Zealand; 6 Department of Medicine, University of Otago, Christchurch 8011, New Zealand; 7 Division of Global and Tropical Health, Menzies School of Health Research, Charles Darwin University, Darwin, NT 0811, Australia; 8 Department of Health, Culion Sanatorium and General Hospital, Culion 5315, Philippines; 9 Division of Programmes for Disease Control, Manila 1003, Philippines

**Keywords:** leprosy, feasibility studies, single-dose rifampicin, *Mycobacterium leprae*, household contacts, post-exposure prophylaxis

## Abstract

Kiribati is a Pacific Island nation with a widely dispersed population and one of the highest rates of leprosy worldwide. Single-dose rifampicin post-exposure prophylaxis (SDR-PEP) of leprosy contacts has reduced new case detection rates in controlled trials. In 2018, an SDR-PEP programme was introduced in Kiribati that included screening and chemoprophylaxis of household contacts of leprosy cases retrospectively (2010–2017) and prospectively (2018–2022). We conducted a retrospective audit to determine the comprehensiveness, timeliness and feasibility of the SDR-PEP programme. Overall, 13,641 household contacts were identified (9791 in the retrospective and 3850 in the prospective cohort). In the retrospective cohort, 1044 (11%) contacts were absent, 403 (4%) were ineligible for SDR, and 42 new cases were detected (0.4%) Overall, SDR coverage was 84.7%. In the prospective cohort, 164 (4%) contacts were absent, 251 (7%) were ineligible for SDR, and 23 new cases were diagnosed (0.6%). Overall, SDR coverage was 88.1%. Across both cohorts, there were 23 SDR refusals. The median time to SDR administration was 220 days (IQR 162–468) and 120 days (IQR 36–283) for the retrospective and prospective cohorts, respectively. SDR was readily accepted in both cohorts. The new case detection rate (0.5%) is consistent with that in other studies. Overall SDR coverage in both the retrospective and prospective phases met programmatic expectations.

## 1. Introduction

Kiribati faces severe leprosy control challenges because of its widely dispersed population, spread across 33 small atolls in the Pacific Ocean. The leprosy new case detection rate in Kiribati is one of the highest in the world, with 11.5 cases per 10,000 population reported in 2022 [1]. The WHO target for leprosy elimination as a public health problem of <1 prevalent case per 10,000 people per year was reached briefly in 2000 following a mass population screening and chemoprophylaxis programme (single-dose rifampicin, ofloxacin and minocycline) in 1997 and 1998 [2]. However, the impact of this intervention was not sustained, and the number of new cases and child cases increased during a period of passive surveillance between 1999–2009. Because of an increasing number of cases, an intensified awareness programme was implemented in 2011, and an active case-finding programme was commenced in 2016, which included skin camps, contact tracing and screening of household contacts [2]. Active case-finding did not extend to neighbours or social contacts. 

Single-dose rifampicin (SDR) post-exposure chemoprophylaxis (SDR-PEP) has been shown to reduce the risk of new incident leprosy amongst leprosy contacts by 57% at 2 years, with added benefit in those who previously received BCG vaccination [3]. Those most at risk of leprosy are household contacts, although neighbours and social contacts are also at increased risk compared with the general population. Mathematical modelling was used to estimate the possible benefit of various chemoprophylaxis approaches in Kiribati [4]. This predicted that the introduction of household contact (HHC) chemoprophylaxis would lead to a gradual but sustained reduction in the number of new leprosy cases. More than 80% of I-Kiribati people receive the BCG vaccination at birth, and therefore the efficacy of SDR-PEP in Kiribati was predicted to be strong. 

After considering this evidence, the Ministry of Health and Medical Services (MH&MS) of Kiribati adopted SDR for household contacts as a policy in 2017 and partnered with the Pacific Leprosy Foundation (PLF) to implement the chemoprophylaxis programme, alongside pre-existing active case-finding activities, in 2018. After careful consideration of both the potential intensity of contacts’ leprosy exposure and the privacy of the index patient, a HHC was defined as a nuclear or extended family member who used the same kitchen as the index patient. Due to the high population density and the complexity of social networks in Kiribati (for example, due to the frequent use of village ‘maneaba’ (community buildings) for meetings, ceremonies and religious and educational activities), chemoprophylaxis of social contacts was not feasible as part of this intervention but was planned as part of a population-wide mass chemoprophylaxis programme when resources allowed. The chemoprophylaxis programme began in 2018 and consisted of retrospective (catch-up) screening and SDR chemoprophylaxis of HHCs of new leprosy cases diagnosed between 2010 and 2017 (retrospective cohort), as well as ongoing prospective screening and chemoprophylaxis of HHCs of new leprosy cases diagnosed from 2018 onwards (prospective cohort). The screening and case-finding were integrated into routine community clinical services and coordinated centrally by the National Leprosy Programme. 

Since the initiation of the chemoprophylaxis programme in Kiribati, further studies have demonstrated the feasibility and acceptability of contact tracing and SDR chemoprophylaxis [5], and the approach has been endorsed by the World Health Organization (WHO) [6]. Pillar 2 of the Global Leprosy Strategy 2021–2030 focuses on scaling up leprosy prevention alongside integrated active case detection to break the chain of transmission [7]. 

The objective of this study was to evaluate the feasibility of the chemoprophylaxis programme in terms of the completeness and timeliness of contact tracing and SDR-PEP administration, as well as the acceptability of the SDR chemoprophylaxis program across all health districts in Kiribati—a geographically widely dispersed and resource-poor setting. A target of 80 per cent coverage was regarded as the minimum to justify the programme [4]. 

## 2. Materials and Methods

This was a retrospective audit using routinely collected data on leprosy patients and contacts recorded in the electronic database of the National Leprosy Programme (NLP) in Kiribati. 

### 2.1. Setting

The National Leprosy Programme is located in the skin clinic at the base hospital in Nawerewere on South Tarawa, the most populous island in Kiribati (population approximately 63,439) [8,9]. The staff includes a doctor with a postgraduate qualification in dermatology, a medical assistant and four specialist nurses. There are also 115 community clinics in Kiribati staffed by nurses and medical assistants who were involved in the programme. All suspected leprosy cases from South Tarawa and the Outer Islands of Kiribati are referred to the NLP for validation, complex case management and maintenance of clinical records. Leprosy cases are classified according to WHO criteria [6]. In brief, paucibacillary (PB) disease is defined as a case with fewer than 5 skin lesions and multibacillary (MB) disease as a case with 5 or more lesions. 

The case-finding strategy during the 2010–2015 period was largely passive, with limited active case-finding occurring in the form of school surveys. Active case-finding increased from 2015 onwards in a non-systematic manner in the form of skin camps and increased school surveys, with active screening of HHCs introduced systematically in 2016. These active case-finding strategies continued over the prospective study period and were enhanced with the introduction of twice-weekly skin clinics from 2018 onwards.

All primary care following case confirmation is delivered through village clinics located on the inhabited atolls. Community health services are divided into geographically related districts that each include multiple inhabited atolls. Routine care is integrated into the primary care clinical services run by medical assistants and nurses who are responsible for passive case detection, screening of HHCs, implementation of the SDR-PEP programme, provision of multidrug treatment packs and referral of patients with complications to the NLP. 

An experienced leprologist (AC) visits South Tarawa regularly to conduct education sessions for medical assistants and nurses at the NLP and contact tracers. He also reviews and validates leprosy cases on South Tarawa. The specialist nurses from the NLP visit the outer islands regularly to conduct education of local nurses, validate cases and review the SDR-PEP programme. 

### 2.2. Data Collection 

The NLP records the following data on physical data collection forms: patient registration number for both cases and contacts, name, address, current location of residence, date of diagnosis, treatment history, clinical data and date and dose of SDR, if received. The hard copy data entry forms are scanned and sent monthly to the PLF office in Christchurch, New Zealand, where the information is entered into a secure Microsoft Access database. The database is used to generate lists of household contacts by index case and village to facilitate the planning of contact tracing activities and workforce allocation. 

### 2.3. SDR Chemoprophylaxis Programme 

The SDR-PEP programme was integrated into the services provided by general community clinics in 2018. After validation or consultation, the medical clinics are notified of a case, and the staff are responsible for following up the cases, enumeration of the households, administering SDR-PEP and reporting data back via a standardised report form to the NLP. Index cases diagnosed from 2010–2017 were identified from the database for the retrospective component and notified to the appropriate clinic. New cases identified prospectively from 2018–2022 were notified to clinics at the time of diagnosis. 

Each village medical clinic was supported to perform promotional activities to raise awareness of the programme, complemented by nationwide initiatives including the use of national radio and social media. Clinic nurses were trained in the diagnosis of leprosy and, with support from the NLP, set up contact tracing teams in each location. Nursing staff visited the residence of the index case, verified the accuracy of the leprosy diagnosis, sought consent to trace and screen all current and previous members and enumerated the household contacts. Household contacts of index patients were traced and screened at their households. Contacts were examined for signs of leprosy, and any suspected cases were referred to the NLP for confirmation or exclusion of the diagnosis by a leprosy specialist. The remainder were given SDR immediately, except contacts of newly diagnosed patients for whom SDR administration was postponed for 1 month after MDT initiation. Absentees were documented and followed up at a later date by the nursing team.

### 2.4. Inclusion Criteria

Household contacts were defined as all those family members sharing the same kitchen facilities as the index case. This included all those living in the household for more than 30 days at any time in the past 2 years. For the prospective component, HHCs were identified at the time of diagnosis of a new leprosy case. 

### 2.5. Exclusion Criteria for SDR PEP

The following rendered HHCs ineligible for SDR-PEP: current TB or leprosy treatment, pregnancy, age < 2 years, history of serious liver or kidney disease, severe medical illness requiring hospitalisation, terminal illness, known allergy to rifampicin or prior severe adverse effect with rifampicin use. 

### 2.6. Chemoprophylaxis Regimen

All contacts of leprosy cases meeting eligibility criteria were offered SDR as chemoprophylaxis. Single-dose rifampicin dosing was based on age and weight (10 mg/kg), and the dosing regimens are outlined in the Supplementary Material (Table S1).

### 2.7. Audit Procedures 

A systematic audit of electronic records for both the retrospective and prospective SDR-PEP cohorts held in the NLP database was performed. Index cases were cross-referenced with household contact information to ensure the removal of duplicates. The primary outcome measures included the proportion of retrospective and prospective contacts traced, SDR coverage in both cohorts, time to SDR administration and SDR refusal rate. The time to delivery of SDR-PEP for the retrospective component was defined as the interval between introduction of the programme on 1 January 2018 and administration of the SDR-PEP dose, and in the prospective cohort, the time interval from diagnosis of the index case to administration of the SDR-PEP dose to contacts.

### 2.8. Data Analysis

The analyses presented are descriptive. Continuous variables are summarised using means, medians, standard deviations, ranges and interquartile ranges as appropriate. Categorical variables are summarised with frequencies and percentages. Categorical variables were compared using Chi-squared tests. A *p*-value less than 0.05 was considered statistically significant. Analyses were done in Microsoft Excel 2010.

### 2.9. Ethical Committee Approval

Ethical approval was obtained from the Kiribati Ministry of Health and Medical Services, the University of Otago (H22/111) and the University of Sydney (project no. 2021/127).

## 3. Results

**Figure 1:**
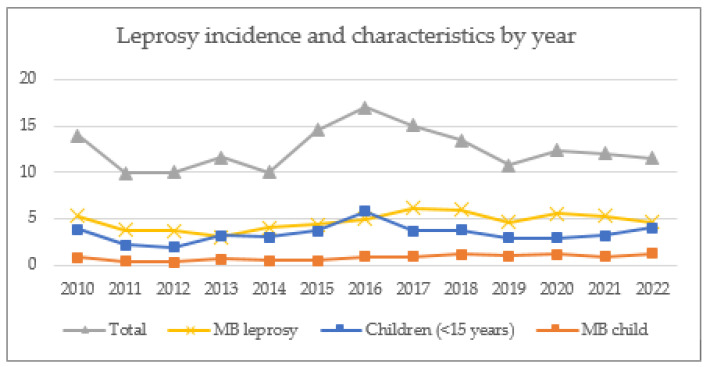
Leprosy incidence (per 10,000 population) and characteristics by year.

The annual incidence of new leprosy cases and disease characteristics over the study period are presented in Table 1. The case detection rate peaked in 2016 at 202 cases per 10,000 population per year, with a gradual decline in overall incidence in subsequent years. The rates of MB leprosy and childhood cases remained high at the end of the study period, accounting for 40% and 35% of cases in 2022, respectively (Figure 1). The characteristics of index patients from the five main health districts for both study cohorts are presented in Table 2. There were 1173 cases identified in the retrospective cohort, of whom 48% were female, and 34% were classified as having MB leprosy. Four per cent of patients had a grade 2 disability at presentation, and 27% were children under the age of 15 years. In the prospective cohort, 762 index cases were identified, of whom 46% were female, and 43% were classified as having MB leprosy. Three per cent had a grade 2 disability, and 28% were children under the age of 15 years. 

Overall, there were 13,641 household contacts of index patients identified during the study period (9791 in the retrospective and 3850 in the prospective cohort). Of these, 1044 HHCs were either absent or unable to be traced in the retrospective cohort and 164 in the prospective cohort, leaving 8747 contacts who were screened in the retrospective and 3688 in the prospective cohort. The number of HHCs in each health district, their screening status and reasons for exclusion from SDR-PEP are presented in Table 3. 

In the retrospective cohort, 8297 received SDR-PEP, representing 95% of contacts screened and 84.7% overall SDR coverage of all contacts. The most common reasons for exclusion from chemoprophylaxis in this group were active or former leprosy (40%) and being less than 2 years of age (32%). Four patients (1%) refused chemoprophylaxis. There were 42 cases of leprosy diagnosed as a result of household contact screening in the retrospective cohort, representing 0.5% of those screened. Of these, 36% were classified as MB and 36% were children less than 15 years of age. There were no reported cases of grade 2 disability in this group (Table 3). 

In the prospective cohort, 3392 contacts received SDR-PEP, representing 92% of contacts screened and 88.1% overall SDR coverage of all enumerated contacts. The most common reasons for exclusion from SDR-PEP were being less than 2 years of age (50%), followed by pregnancy (15%) and active or former leprosy (14%). Nineteen patients (8%) in the prospective cohort refused chemoprophylaxis. There were 23 new diagnoses of leprosy as a result of screening of household contacts in the prospective cohort (0.6% of those screened). Of these, 7 (30%) were classified as MB, 2 (9%) had grade 2 disability, and 6 (26%) were children under the age of 15 (Table 3).

The median time to administration of SDR-PEP for each of the health districts over the study period is presented in Table 3. In the retrospective cohort, the median time between the intervention start date and receiving SDR-PEP was 220 days (interquartile range [IQR] 162–468 days). The median time to SDR from index case diagnosis in the retrospective cohort was 1311 days (IQR 777–2105 days). Those closer to larger population centres received prophylaxis sooner than more geographically isolated contacts, such as those in the outer islands. In the prospective cohort, the median time to receive SDR-PEP was 120 days (IQR 36–283 days). The percentage of cases of PB in children decreased after the introduction of the SDR-PEP programme (2018–2022) compared with the retrospective study period (2010–2017), (mean 81% vs. 69%, *p* = 0.0002), with a corresponding increase in child MB cases (mean 19% vs. 31%, *p* = 0.0002).

## 4. Discussion

The aim of this audit was to determine whether it was feasible to integrate an active screening and chemoprophylaxis programme into routine clinical leprosy services in a resource-poor setting with a widely dispersed population in the Pacific. This programme was adopted as a policy in 2017 and implemented in 2018 by the MH&MS of Kiribati as a tool to improve leprosy control in response to the very high new case detection rate following the introduction of active case-finding in 2016. Overall, we found that >85% of HHCs were traced in both the retrospective and prospective cohorts, a significant number of new cases were identified on screening, and a very high acceptance rate of SDR-PEP was observed. However, there were significant delays to SDR administration, largely because of the widely dispersed population. 

Retrospective contact tracing and SDR-PEP were undertaken as part of the LPEP study, but to our knowledge, only reported specifically in the Cambodian cohort, a country with a much greater population and significantly lower leprosy endemicity than Kiribati [5,11]. The average number of contacts per index case screened in the Cambodian study was higher than in Kiribati (19 vs. 7), but the proportion of contacts screened was lower (72% vs. 91%), and the number of exclusions was significantly higher (17.4% vs. 5%) in Cambodia compared to Kiribati. The number of new cases detected amongst HHCs in Cambodia (0.4% of those screened, 1/3 of whom were neighbours) was similar to that in Kiribati (0.5%). These results demonstrate that the retrospective approach provides useful gains in terms of case detection and can be used as part of an enhanced control strategy in both low- and high-endemic settings. 

Overall, the programme in Kiribati screened 12,435 contacts, which represents 10% of the total population. After exclusions, SDR-PEP was administered to 8297 and 3392 contacts in these cohorts, respectively. The proportion of contacts identified and screened was lower in the retrospective cohort than in the prospective one. The primary reason for this was the high number of people who moved away from the area where they had been a HHC in the retrospective cohort. Most of these HHCs had moved within Kiribati, but it was often not possible to identify their new location as there is no street address system in Kiribati, and it was not practicable to conduct house-to-house enquiries. A smaller group had moved to other countries and were also not traced. Contacts were often not available during daylight hours when people were unavailable because of work, fishing or visiting neighbouring islands.

The very high rate of acceptance of SDR-PEP in both groups is consistent with previous studies that indicated that people with leprosy are very keen to prevent their relatives and contacts from getting the disease [12]. Other factors that may have contributed to this success are the effectiveness of the initial communication and awareness programmes that were conducted by local community health workers who were known to, and trusted by, the populace and the effective communication skills of the nurses doing the screening. Unfortunately, as access to the internet is becoming available in Kiribati, misinformation about leprosy is surfacing. 

The number of HHCs enumerated and traced per index case was lower than expected, particularly in the prospective cohort. The definition used in this programme was much more limited than that proposed by WHO and used in other studies, as it did not include neighbours or social contacts [1,5,13]. This was the case because of concerns about the confidentiality of including extended contacts, given the stigma associated with leprosy [14]. Certain areas of Kiribati are densely populated (for example, in the most populous part of South Tarawa, Betio, approximately 20,000 people live within 1.5 square kilometres). Defining the limits of significant contact exposure in such environments is very difficult. For this reason, a mass SDR-PEP programme was seen as necessary in addition to the contact tracing, and such a programme has now been commenced as part of a large implementation study combining leprosy treatment and mass chemoprophylaxis with enhanced tuberculosis detection, treatment and chemoprophylaxis [15]. The need for such an approach is also supported by the persisting high number of child cases and MB disease in children, which are suggestive of ongoing community transmission. Mapping of cases is currently being done to inform on priority areas for mass chemoprophylaxis.

The wide geographic distribution of inhabited islands across millions of square kilometres of ocean posed a significant barrier to the implementation of the SDR-PEP programme in Kiribati. There were significant delays in the implementation of the policy in some health districts. This is related to the very high costs of maintaining the education, competence and enthusiasm of local staff in remote areas. To improve performance, staff from the NLP visited the outer islands as part of multidisciplinary teams both to support local staff and help with screening contacts and administration of SDR-PEP. Some delays were also attributable to the COVID-19 control measures in 2019–2021, which included lockdowns, interruption of supply of rifampicin and reallocation of staff to other duties. 

The roll-out of the project had additional indirect benefits, as previously reported by others [5]. The SDR-PEP project renewed attention on the high rates of leprosy at the political level, increased leprosy awareness amongst the general population and improved leprosy knowledge of staff at the NLP as well as medical assistants and nurses at the community clinics. 

In conclusion, the SDR-PEP programme integrated into routine community services produced high coverage and acceptability in Kiribati. A centralised database managed through the NLP was pivotal, and additional human resources from community clinics were needed to support contact tracing activities and ensure adequate SDR-PEP coverage. Both the retrospective and prospective components identified a significant number of new cases. This makes an important contribution to leprosy control but needs to be supported with mass chemoprophylaxis, given the high new case detection rate and population density of Kiribati.

## Figures and Tables

**Table 1:** Leprosy incidence and characteristics by year for Kiribati *.

Year	2010	2011	2012	2013	2014	2015	2016	2017	2018	2019	2020	2021	2022
**Population**	107,995	109,871	111,618	113,311	114,985	116,707	118,513	120,362	122,261	124,241	126,463	128,874	131,232
**No. of Cases**	151	109	112	132	115	170	202	182	165	134	157	155	151
**Incidence (per 10,000 Population)**	14.0	9.9	10.0	11.6	10.0	14.6	17.0	15.1	13.5	10.8	12.4	12.0	11.5
**MB leprosy, *n* (%)**	57 (38)	42 (39)	41 (37)	34 (26)	46 (40)	51 (30)	59 (29)	74 (41)	73 (44)	58 (43)	70 (45)	68 (44)	61 (40)
**Child Cases (<15 years of age), *n* (%)**	42 (28)	24 (22)	22 (20)	36 (27)	35 (30)	43 (25)	69 (34)	45 (25)	46 (28)	37 (28)	37 (24)	41 (26)	53 (35)
** MB (%)**	9 (21)	5 (21)	4 (18)	8 (22)	6 (17)	6 (14)	11 (16)	11 (24)	14 (30)	13 (35)	15 (41)	12 (29)	17 (32)
** PB (%)**	33 (79)	19 (79)	18 (82)	28 (78)	29 (83)	37 (86)	58 (84)	34 (76)	32 (70)	24 (65)	22 (59)	29 (71)	36 (68)

* Population data shown are taken from World Bank population estimates and projections (https://databank.worldbank.org/source/population-estimates-and-projections; accessed on 1 November 2023) [10].

**Table 2:** Characteristics of index patients diagnosed since 2010 for the five main health districts.

	2010–2017						2018–2022					
	South Tarawa	Phoenix & Line	Central Kiribati	Northern Kiribati	Southern Kiribati	Total	South Tarawa	Phoenix & Line	Central Kiribati	Northern Kiribati	Southern Kiribati	Total
	N (%)						N (%)					
Population *	56,388	10,503	7683	20,210	15,352	110,136	63,439	11,279	8406	20,806	16,010	119,940
Index Patients	866	54	48	114	91	1173	509	19	61	93	80	762
MB Leprosy	297 (34)	31 (57)	14 (29)	31 (27)	31 (34)	404 (34)	235 (46)	9 (47)	19 (31)	32 (34)	35 (44)	330 (43)
Grade 2 Disability	36 (4)	3 (6)	0	3 (3)	2 (2)	44 (4)	21 (4)	0	0	3 (3)	0	24 (3)
Sex												
Male	455 (53)	30 (56)	23 (48)	56 (49)	41 (45)	605 (52)	280 (55)	11 (58)	35 (57)	38 (41)	47 (59)	411 (54)
Female	410 (47)	24 (44)	25 (52)	58 (51)	50 (55)	567 (48)	229 (45)	8 (42)	26 (43)	55 (59)	33 (41)	351 (46)
Children (<15 years)	231 (27)	12 (22)	12 (25)	35 (31)	26 (29)	316 (27)	128 (25)	5 (26)	23 (38)	27 (29)	31 (39)	214 (28)

* Health district population data obtained from Kiribati National Statistics Office census data for 2015 and 2020 (https://pacificdata.org/data/dataset/spc_kir_2015_phc_v01_m; https://pacificdata.org/data/dataset/spc_kir_2020_phc_v01_m/; accessed 1 November 2023) [8,9].

**Table 3:** Contacts of patients with leprosy for retrospective and prospective cohorts by health district, screening status, reasons for exclusion and characteristics of new cases.

	2010–2017						2018–2022					
	South Tarawa	Phoenix & Line	Central Kiribati	Northern Kiribati	Southern Kiribati	Total	South Tarawa	Phoenix & Line	Central Kiribati	Northern Kiribati	Southern Kiribati	Total
	N (%)						N (%)					
Population *	56,388	10,503	7683	20,210	15,352	110,136	63,439	11,279	8406	20,806	16,010	119,940
Total Listed contacts	7935	397	319	657	483	9791	2929	77	227	325	292	3850
No. of Contacts per Patient (Mean)	9.2	7.4	6.6	5.8	5.3	8.4	5.8	4.1	3.7	3.5	3.6	5.1
Absent/not traced	891 (11)	15 (4)	44 (14)	28 (4)	66 (14)	1044 (11)	137 (5)	2 (3)	5 (2)	19 (6)	1 (0.5)	164 (4)
Moved within Kiribati	-	-	-	-	-	557 (6)	-	-	-	-	-	52 (1)
Overseas	-	-	-	-	-	88 (1)	-	-	-	-	-	14 (0.5)
Total screened	7044 (89)	382 (96)	275 (86)	629 (96)	417 (86)	8747 (89)	2792 (95)	75 (97)	222 (98)	306 (94)	291 (100)	3686 (96)
Age												
- <2 years	168 (2)	9 (0.5)	8 (3)	16 (3)	13 (3)	214 (2)	147 (5)	1 (1)	8 (4)	7 (23)	11 (4)	174 (5)
- 2–14	2233 (32)	139 (36)	103 (37)	259 (41)	162 (39)	2896 (33)	848 (30)	28 (37)	84 (38)	121 (40)	92 (32)	1173 (32)
- 15–24	1538 (22)	69 (18)	42 (15)	99 (16)	53 (13)	1801 (21)	537 (19)	11 (15)	33 (15)	53 (17)	46 (16)	680 (18)
- 25–49	2274 (32)	126 (33)	91 (33)	177 (28)	119 (29)	2787 (32)	916 (33)	28 (37)	68 (31)	74 (24)	86 (30)	1172 (32)
- ≥50	816 (12)	39 (10)	31 (11)	78 (12)	67 (16)	1031 (12)	338 (12)	7 (9)	29 (13)	51 (17)	56 (19)	481 (13)
No Age Documented	15 (0.5)	0	0	0	3 (1)	18 (0.5)	6 (0.5)	0	0	0	0	6 (0.5)
Met Exclusion criteria for SDR-PEP	303 (4)	29 (7)	16 (5)	26 (4)	29 (7)	403 (5)	210 (8)	3 (4)	11 (5)	9 (3)	18 (6)	251 (7)
Reason for Exclusion from SDR-PEP												
- TB	34 (11)	0	0	6 (23)	0	40 (10)	12 (6)	0	1 (9)	0	1 (6)	14 (6)
- Pregnancy	33 (11)	4 (14)	0	4 (15)	1 (3)	42 (10)	29 (14)	1 (33)	1 (9)	3 (33)	3 (17)	37 (15)
- Underage	96 (32)	7 (24)	8 (50)	8 (31)	8 (28)	127 (32)	108 (51)	0	7 (64)	4 (44)	6 (33)	125 (50)
- Other medical reason	20 (7)	2 (7)	1 (6)	0	2 (7)	25 (6)	15 (7)	1 (33)	0	0	3 (17)	19 (8)
- Active/former leprosy	113 (37)	15 (52)	7 (44)	8 (31)	17 (59)	160 (40)	26 (12)	1 (33)	2 (18)	2 (22)	5 (28)	36 (14)
- Refusal	3 (1)	0	0	0	1 (3)	4 (1)	19 (9)	0	0	0	0	19 (8)
- Other	4 (1)	1 (3)	0	0	0	5 (1)	1 (0.5)	0	0	0	0	1 (0.5)
Eligible for SDR	6741 (96)	353 (92)	259 (94)	603 (96)	388 (93)	8344 (95)	2584 (92)	72 (96)	211 (95)	297 (97)	273 (94)	3437 (93)
Received SDR of total screened	6705 (95)	353 (92)	256 (93)	599 (95)	384 (92)	8297 (95)	2544 (91)	72 (96)	210 (95)	296 (97)	270 (93)	3392 (92)
Overall SDR coverage (%)	84.5	88.9	80.3	91.2	79.5	84.7	86.8	93.5	92.5	91.1	92.5	88.1
Median time to SDR in days (IQR)	191 (159–467)	344 (338–346)	326 (273–494)	386 (139–432)	464 (252–513)	220 (162–468)	138 (48–303)	152 (59–327)	15 (0–107)	63 (2–273)	30 (0–330)	120 (36–283)
No. of newly diagnosed cases through contact tracing	15	7	6	7	7	42	12	1	6	1	3	23
- MB leprosy, N (%)	4 (27)	3 (43)	3 (50)	2 (29)	3 (43)	**15 (36)**	4 (33)	-	1 (17)	-	2 (67)	**7 (30)**
- PB leprosy, N (%)	11 (73)	4 (57)	3 (50)	5 (71)	4 (57)	**27 (64)**	8 (66)	1 (100)	5 (83)	1 (100)	1 (33)	**16 (70)**
- Grade 2 disability, N	0	0	0	0	0	**0**	2 (17)	0	0	0	0	**2 (9)**
Sex												
Male, N (%)	7 (47)	4 (57)	4 (66)	2 (29)	4 (57)	**21 (50)**	7 (58)	1 (100)	4 (66)	-	3 (100)	**15 (65)**
Female, N (%)	8 (53)	3 (43)	2 (33)	5 (71)	3 (43)	**21 (50)**	5 (42)	-	2 (33)	1 (100)	0	**8 (35)**
Children (<15 years), N (%)	7 (47)	2 (29)	2 (33)	4 (57)	0	**15 (36)**	3 (25)	0	2 (33)	0	1 (33)	**6 (26)**

* Health district population data obtained from Kiribati National Statistics Office census data for 2015 and 2020 (https://pacificdata.org/data/dataset/spc_kir_2015_phc_v01_m; https://pacificdata.org/data/dataset/spc_kir_2020_phc_v01_m/; accessed 1 November 2023) [8,9].

## Data Availability

Available upon reasonable request.

## Supplementary Materials

The following supporting information can be downloaded at: https://www.mdpi.com/article/10.3390/tropicalmed9030058/s1, Table S1: Single dose rifampicin (SDR) chemoprophylaxis dosing.
