# Supplementary material for: Single-Dose Rifampicin Leprosy Chemoprophylaxis for Household Contacts in Kiribati: An Audit of a Combined Retrospective and Prospective Approach
Source: Trop Med Infect Dis. 2024 Mar 1;9(3):58. doi: 10.3390/tropicalmed9030058 (PMC10976163; doi:10.3390/tropicalmed9030058)
Supplement: Supplementary file 1 [file tropicalmed-09-00058-s001.zip › tropicalmed-2814846-supplementary.pdf]

Tropical Medicine and Infectious Disease

MDPI

Article

Single-Dose Rifampicin Leprosy Chemoprophylaxis for Household Contacts in Kiribati: An Audit of a Combined Retrospective and Prospective Approach

Patrick O. Campbell [1,2,*,†], Temea Bauro [3,†], Erei Rimon [3], Eretii Timeon [3], Caitlin Bland [4], Nabura Ioteba [5], Nicholas M. Douglas [2,6,7], Arturo Cunanan [8,9] and Stephen T. Chambers [1]

[1] Department of Pathology and Biomedical Science, University of Otago, Christchurch 8011, New Zealand; steve.chambers@otago.ac.nz
[2] Department of Infectious Diseases, Christchurch Hospital, Te Whatu Ora Waitaha, Canterbury 8011, New Zealand; nick.douglas@otago.ac.nz
[3] Government of the Republic of Kiribati Ministry of Health and Medical Services, Tarawa P.O. Box 268, Kiribati; temea.bauro@mhms.gov.ki (T.B.)
[4] Otago Medical School, University of Otago, Christchurch 8011, New Zealand
[5] Pasifika Medical Association, Christchurch 8011, New Zealand
[6] Department of Medicine, University of Otago, Christchurch 8011, New Zealand
[7] Division of Global and Tropical Health, Menzies School of Health Research, Charles Darwin University, Darwin, NT 0811, Australia
[8] Department of Health, Culion Sanatorium and General Hospital, Culion 5315, Philippines
[9] Division of Programmes for Disease Control, Manila 1003, Philippines
* Correspondence: patrick.campbell@cdhb.health.nz
† These authors contributed equally to this work.

Table S1. Single dose rifampicin (SDR) chemoprophylaxis dosing.

Age/Body Weight	Rifampicin Single Dose
>15 years old	600 mg
10–14 years	450 mg
Children 6–9 years (weight ≥ 20 kg)	300 mg
Children 6–9 years (weight < 20 kg)	150 mg
Children < 5 years	10–15 mg/kg

Disclaimer/Publisher's Note: The statements, opinions and data contained in all publications are solely those of the individual author(s) and contributor(s) and not of MDPI and/or the editor(s). MDPI and/or the editor(s) disclaim responsibility for any injury to people or property resulting from any ideas, methods, instructions or products referred to in the content.
